# Early life stress induces long-term changes in limbic areas of a teleost fish: the role of catecholamine systems in stress coping

**DOI:** 10.1038/s41598-018-23950-x

**Published:** 2018-04-04

**Authors:** Marco A. Vindas, Stefanos Fokos, Michail Pavlidis, Erik Höglund, Sylvia Dionysopoulou, Lars O. E. Ebbesson, Nikolaos Papandroulakis, Catherine R. Dermon

**Affiliations:** 1 Uni Environment, Uni Research AS, Bergen, Norway; 2 Institute of Neuroscience and Physiology, University of Gothenburg, Gothenburg, Sweden; 3 Department of Biology, Human and Animal Physiology Lab, University of Patras, Heraklion, Greece; 4 Department of Biology, University of Crete, Heraklion, Greece; 5 National Institute of Aquatic Resources, Technical University of Denmark, Hirtshals, Denmark; 6 Norwegian Institute for Water Research (NIVA), Oslo, Norway; 7 Institute of Marine Biology, Biotechnology and Aquaculture, Hellenic Centre for Marine Research, Heraklion, Greece

## Abstract

Early life stress (ELS) shapes the way individuals cope with future situations. Animals use cognitive flexibility to cope with their ever-changing environment and this is mainly processed in forebrain areas. We investigated the performance of juvenile gilthead seabream, previously subjected to an ELS regime. ELS fish showed overall higher brain catecholaminergic (CA) signalling and lower brain derived neurotrophic factor (*bdnf*) and higher *cfos* expression in region-specific areas. All fish showed a normal cortisol and serotonergic response to acute stress. Brain dopaminergic activity and the expression of the α_2Α_ adrenergic receptor were overall higher in the fish homologue to the lateral septum (Vv), suggesting that the Vv is important in CA system regulation. Interestingly, ELS prevented post-acute stress downregulation of the α_2Α_ receptor in the amygdala homologue (Dm3). There was a lack of post-stress response in the β_2_ adrenergic receptor expression and a downregulation in *bdnf* in the Dm3 of ELS fish, which together indicate an allostatic overload in their stress coping ability. ELS fish showed higher neuronal activity (*cfos*) post-acute stress in the hippocampus homologue (Dlv) and the Dm3. Our results show clear long-term effects on limbic systems of seabream that may compromise their future coping ability to environmental challenges.

## Introduction

Animals cope with stressful situations in different manners. Generally, an interaction between genes and both current and past environments gives rise to these individual variations in stress coping. In this context, early life experiences may shape the way individuals cope with stress in future situations^1–4^. In this way, stressful stimuli over prolonged periods of time (*i*.*e*. chronic stress), have often been associated with expression of maladaptive behaviour and neurobiological diseases^5^. Furthermore, experiencing chronic stress during early life stages (including adolescence), may lead to long-term physiological and behavioural changes which may not be adaptive to a current non-stressful environment. Potentially, this leads to normally adaptive responses over-riding self-correcting tendencies of emotional mechanisms, leading to pathologies^6–9^.

Animals have to balance attention, inhibition of active behaviour and cognitive processing in relation to internal and external feedback in an ever-changing environment^10^. The processing of stimuli is under top-down control from forebrain areas, such as the cortex and limbic areas^10,11^. For example, the amygdala is of great importance in the evaluation of emotional stimuli salience^11^. Accordingly, in mammals it has been proposed that the hippocampus and particularly both the amygdala and lateral septum are the main regulators that mediate the long-term effects of early life stress (ELS) on behavioural outcomes, such as anxiety-like behaviour^9,12,13^. In contrast to mammals, the fish’s telencephalon lacks a 6-layered pallium (*i*.*e*. they do not possess a cortex). However, the lack of a cortex does not imply an absence of so-called “higher functions”, and telencephalic cortical-like functions have been reported in several fish species^14–21^. The fish’s telencephalon contains several distinct neuronal populations, which have been characterized as functional homologues to mammalian forebrain areas. For example, the dorsomedial (Dm) and dorsolateral (Dl) pallium have been characterised as functional homologues to the mammalian cortical (basolateral) amygdala and hippocampus, respectively, and are implicated in stimuli salience, memory and learning^22–24^. Furthermore, the ventral part of the ventral telencephalon (Vv) has been reported to be functionally homologous to the lateral septum^22,23^, which is very important in encoding stimuli salience and emotional coding and is associated with the regulation of emotional reactivity and goal-oriented behaviour^25,26^.

Here we explore the neurobiological long-term effects of an ELS regime applied in gilthead seabream (*Sparus auratus*) in subsequent juvenile development stages. We hypothesize that ELS alters neurobiological systems that potentially affects how individuals cope with their environment. Specifically, we analysed the plasma cortisol response, the brain monoamine neurochemistry and the neural expression of adrenergic receptors at basal and acute-stress conditions. In addition, we quantified the expression of the neuronal activity marker *cfos* and the neuroplasticity marker brain derived neurotrophic factor (*bdnf*) at basal and post-stress conditions. We report for the first time, stress resilient mechanisms involving catecholamine and region-specific adrenergic receptor expression in fish. Notably, this is in agreement with mammalian literature on effects of ELS on catecholamine function, particularly in terms of lateral septum and amygdala function^9,27^, which highlights the relevance of using fish models in the study of central nervous system function in vertebrates.

## Results

We found long-term effects on seabream limbic systems sampled at either basal levels or after an acute stress test, approximately 5 months after being subjected to an early life stress (ELS) regime for 2 weeks. Note that this ELS was conducted at either the first feeding stage (FF) or at the formation of all fins to melanophores stage (AF).

### Cortisol

**Figure 1:**
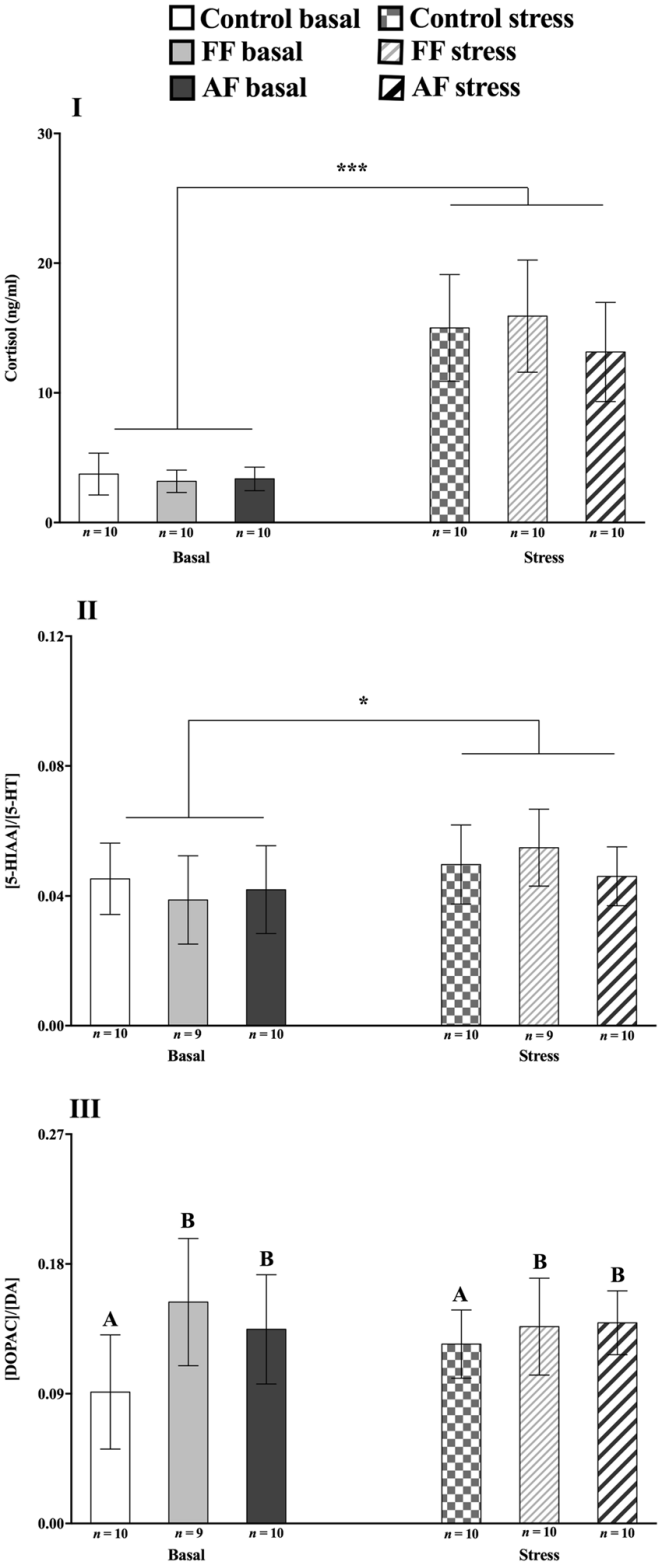
Cortisol and monoamine neurochemistry at basal and post-stress conditions. Effect of treatment (control *vs*. early life stress regime at first feeding stage; FF and at all fins stage; AF) and conditions (basal *vs*. acute stress) on mean (±SEM) cortisol concentrations (**I**), the 5-HIAA/5-HT (**II**) and the DOPAC/DA (**III**) ratios. Where * indicates an acute stress effect and capital letters a treatment effect. ANOVA Statistics for Cortisol: Treatment, *F*_(3,56)_ = 0.61, *p* = 0.55 and Conditions: *F*_(3,56)_ = 392, *p* < 0.001. For 5-HIAA/5-HT: Treatment, *F*_(3,54)_ = 0.47, *p* = 0.63 and Conditions: *F*_(3,54)_ = 6.3, p = 0.02. For DOPAC/DA: Treatment, *F*_(3,55)_ = 5.9, *p* = 0.005 and Conditions: *F*_(3,55)_ = 0.64, *p* = 0.43.

There was a significant increase of cortisol levels post-acute stress in all groups (*p* < 0.001) but there was no significant treatment or interaction effects (Fig. 1I).

### Monoamine neurochemistry

#### Serotonergic neurochemistry

There was a significant post-acute stress increase in 5-HIAA concentrations (*p* = 0.002; Table S1) and in 5-HIAA/5-HT ratios (*p* = 0.02), with no significant treatment or interaction effects found for these parameters (Fig. 1II). No significant effects were found for 5-HT concentrations (Table S1).

#### Dopaminergic neurochemistry

There was a treatment (*p* = 0.009) and an interaction (*p* = 0.04) effect in DOPAC concentrations, with FF fish showing higher basal levels (*p* = 0.003), and AF higher post-acute stress levels (*p* = 0.02), compared to control fish at basal conditions (Table S1). In addition, the DOPAC/DA ratios were overall higher in ELS fish, compared to control fish (*p* = 0.005; Fig. 1III), No significant treatment or interaction effects were found for these parameters and no significant effects were found for DA concentrations (Table S1).

#### Noradrenaline neurochemistry

There were no significant effects on NA concentrations (Table S1).

### Adrenergic receptors

#### α_2Α_

**Figure 2:**
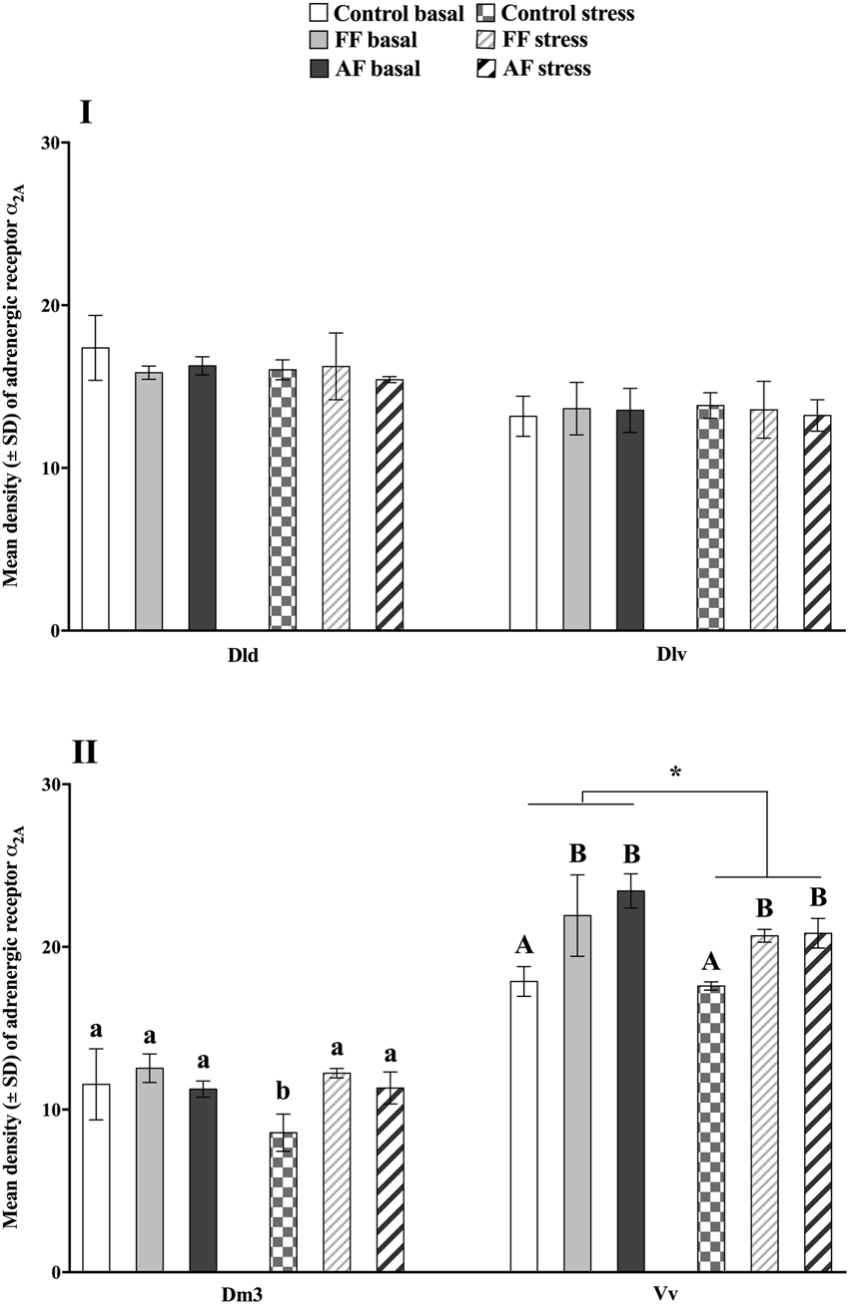
Adrenergic receptor (α_2A_) expression at basal and post-stress conditions. Effect of treatment (control *vs*. early life stress regime at first feeding stage; FF and at all fins stage; AF), conditions (basal *vs*. acute stress) and the interaction between treatment and conditions (if it was maintained in the model which was indicated by “lack of fit” analysis) on mean (±SEM) density of the adrenergic receptor α_2A_ in the dorsal (Dld) and ventral (Dlv) parts of the dorsolateral pallium (**I**), the dorsomedial pallium subdivision 3 (Dm3) and the ventral part of the ventral telencephalon (Vv; **II**). Capital letters indicate a treatment effect, small letters indicate an interaction effect and * indicates an acute stress effect. ANOVA Statistics for the Dld Treatment, *F*_(2,18)_ = 1.1, *p* = 0.35 and Conditions: *F*_(2,18)_ = 1.63, *p* = 0.22. Dlv Treatment, *F*_(2,18)_ = 0.06, *p* = 0.94 and Conditions: *F*_(2,18)_ = 0.03, *p* = 0.86. Dm3 Treatment, *F*_(5,18)_ = 8, *p* = 0.003, Conditions: *F*_(5,18)_ = 5.1, *p* = 0.04 and Interaction: *F*_(5,18)_ = 4.1, *p* = 0.04 and the Vv Treatment, *F*_(2,18)_ = 26, *p* < 0.001 and Conditions: *F*_(2,18)_ = 6.9, *p* = 0.02.

No significant effects were found in the dorsal (Dld) or ventral (Dlv) dorsolateral pallium (Fig. 2I). However, there were both significant treatment (*p* < 0.001) and conditions (*p* = 0.02) effects in the ventral part of the ventral telencephalon (Vv), with both AF and FF groups showed overall higher α_2Α_ expression compared to control fish and there was an overall reduction in post-acute stress receptor expression in all groups (Fig. 2II). In addition, there was a significant interaction effect in the dorsomedial pallium subdivision 3 (Dm3), with control post-acute stress groups showing the lowest receptor expression values as compared to all other groups (Fig. 2II).

#### β_2_

**Figure 3:**
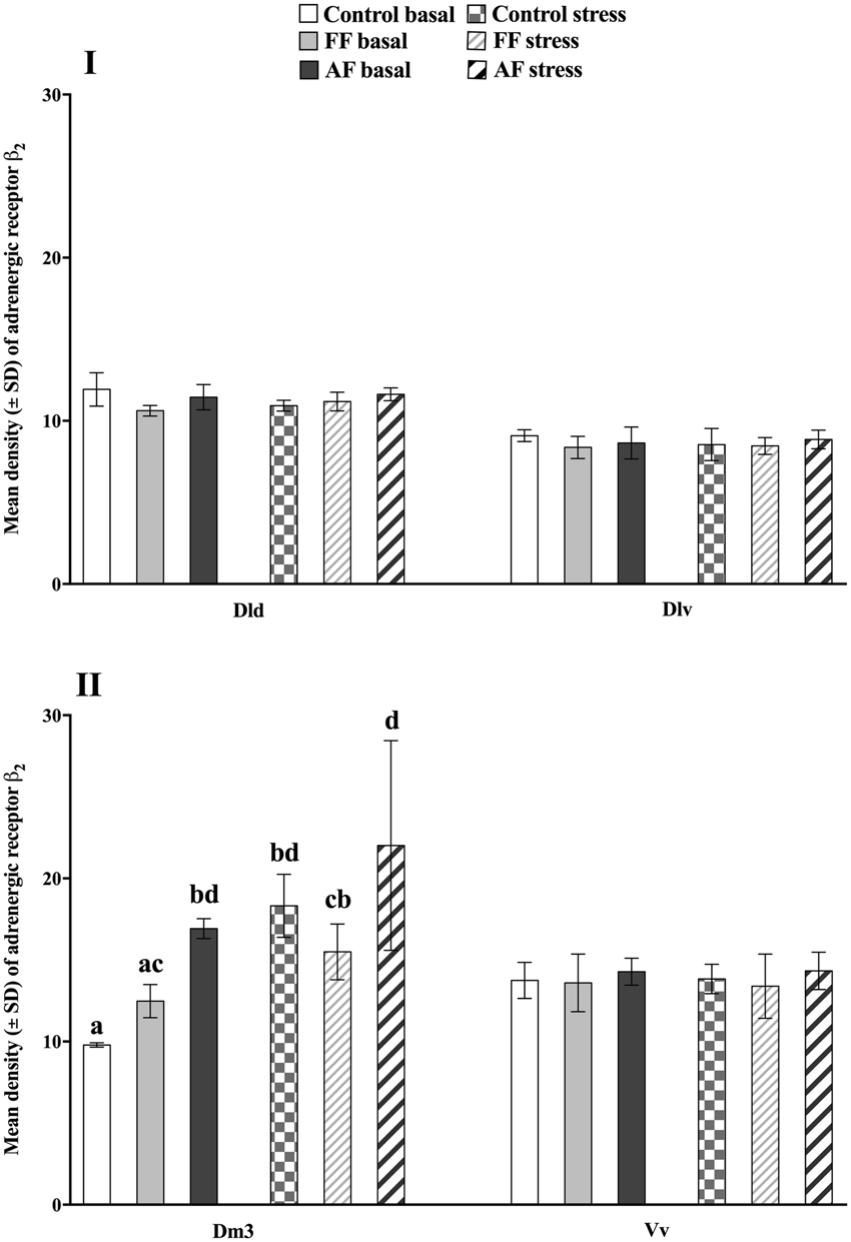
Adrenergic receptor (β_2_) expression at basal and post-stress conditions. Effect of treatment (control *vs*. early life stress regime at first feeding stage; FF and at all fins stage; AF), conditions (basal *vs*. acute stress) and the interaction between treatment and conditions (if it was maintained in the model which was indicated by “lack of fit” analysis) on mean (±SEM) density of the adrenergic receptor β_2_ in the dorsal (Dld) and ventral (Dlv) parts of the dorsolateral pallium (**I**), the dorsomedial pallium subdivision 3 (Dm3) and the ventral part of the ventral telencephalon (Vv; **II**). Small letters indicate an interaction effect. ANOVA Statistics for Dld Treatment, *F*_(2,18)_ = 1.1, *p* = 0.17 and Conditions: *F*_(2,18)_ = 0.09, *p* = 0.76. Dlv Treatment, *F*_(2,18)_ = 0.75, *p* = 0.45 and Conditions: *F*_(2,18)_ = 0.08, *p* = 0.78. Dm3 Treatment, *F*_(5,18)_ = 15.9, *p* < 0.001, Conditions: *F*_(5,18)_ = 40.1, *p* < 0.001 and Interaction: *F*_(5,18)_ = 5.7, *p* = 0.01. Vv Treatment, *F*_(2,18)_ = 0.81, *p* = 0.46 and Conditions: *F*_(2,18)_ = 0.002, *p* = 0.96.

There was a significant treatment (*p* < 0.001), conditions (*p* < 0.001) and interaction (*p* = 0.01) effect in the Dm3, were the basal control group displayed a lower receptor expression compared to all other groups, except for the basal FF group. In addition, the basal FF group had a significantly lower receptor expression compared to basal and post-acute stress AF as well as the post-acute stress control groups (Fig. 3II). No other significant effects were found in the remaining areas for the β_2_ receptor expression (Fig. 3).

### ISH of *cfo*s and *bdnf* labelled cells

#### bdnf

**Figure 4:**
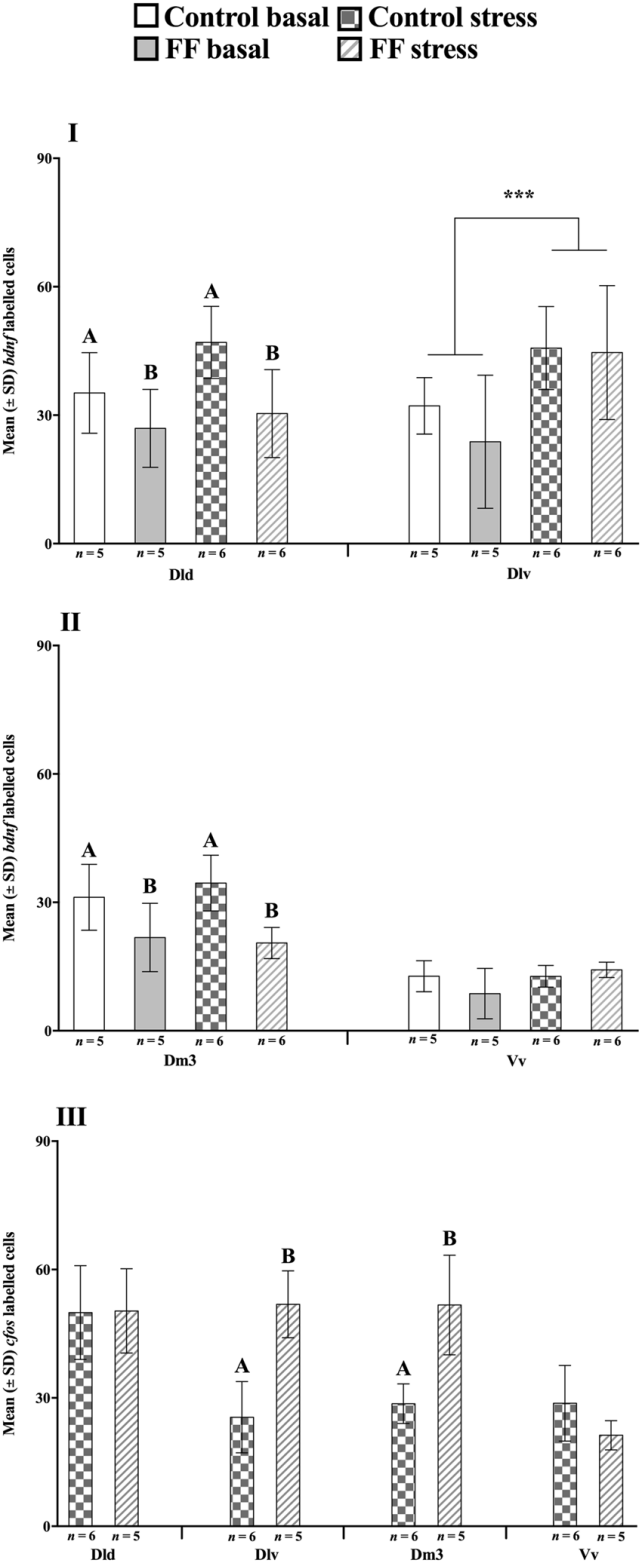
*In situ* hybridization labelled *bdnf* and *cfos* cells. Effect of treatment (control vs. early life stress regime at first feeding stage; FF) and conditions (basal vs. acute stress) on mean (±SD) expression of brain derived neurotrophic factor (*bdnf*; **I**, **II**) and *cfos* (**III**) labelled cells in the dorsal (Dld) and ventral (Dlv) parts of the dorsolateral pallium, the dorsomedial pallium subdivision 3 (Dm3) and the ventral part of the ventral telencephalon (Vv). Capital letters indicate a treatment effect and * indicates an acute stress effect. ANOVA Statistics for *bdnf*: Dld Treatment, *F*_(2,19)_ = 10.3, *p* = 0.005 and Conditions: *F*_(2,19)_ = 3.6, *p* = 0.07 and Dlv Treatment: *F*_(2,19)_ = 0.69, *p* = 0.4. Conditions: *F*_(2,19)_ = 10.5, *p* = 0.004. Dm3 Treatment, *F*_(2,19)_ = 18.5, *p* < 0.001 and Conditions: *F*_(2,19)_ = 0.13, *p* = 0.72 and Vv Treatment: *F*_(2,18)_ < 0.001, *p* = 0.98. Conditions: *F*_(2,18)_ = 1.9, *p* = 0.18. Students *t* test statistics for *cfos*: Dld *t*_(8.9)_ = 0.06, *p* = 0.95, Dlv *t*_(8.8)_ = 5.4, *p* < 0.001, Dm3 *t*_(5)_ = 4.2, *p* = 0.009 and Vv *t*_(6.7)_ = −1.9, *p* = 0.1. Data are presented as mean ± SEM.

ISH analysis of *bdnf* transcript abundance showed clear post-acute stress activation in the Dlv (*p* = 0.004), with both FF and control groups showing a post-acute stress increase in *bdnf* expression (Fig. 4I). In addition, there was a treatment effect in both the Dld (*p* = 0.005; Fig. 4I) and the Dm3 (*p* < 0.001, Fig. 4II), were control groups showed an overall higher *bdnf* abundance compared to FF groups. No other significant effects were found for *bdnf* transcript abundance.

#### cfos

Basal levels of *cfos* transcript abundance were not detectable, probably due to the fact that all ISH samples (*i*.*e*. basal and post-stress) were processed together and we had to stop the colouring reaction before any cells were clearly labelled under basal conditions to avoid background staining in post-acute stress samples. There was a significant difference between control and FF groups post-acute stress in the Dlv (*p* < 0.001) and the Dm3 (*p* = 0.009), were FF groups showed a higher *cfos* abundance (Fig. 4III).

**Figure 5:**
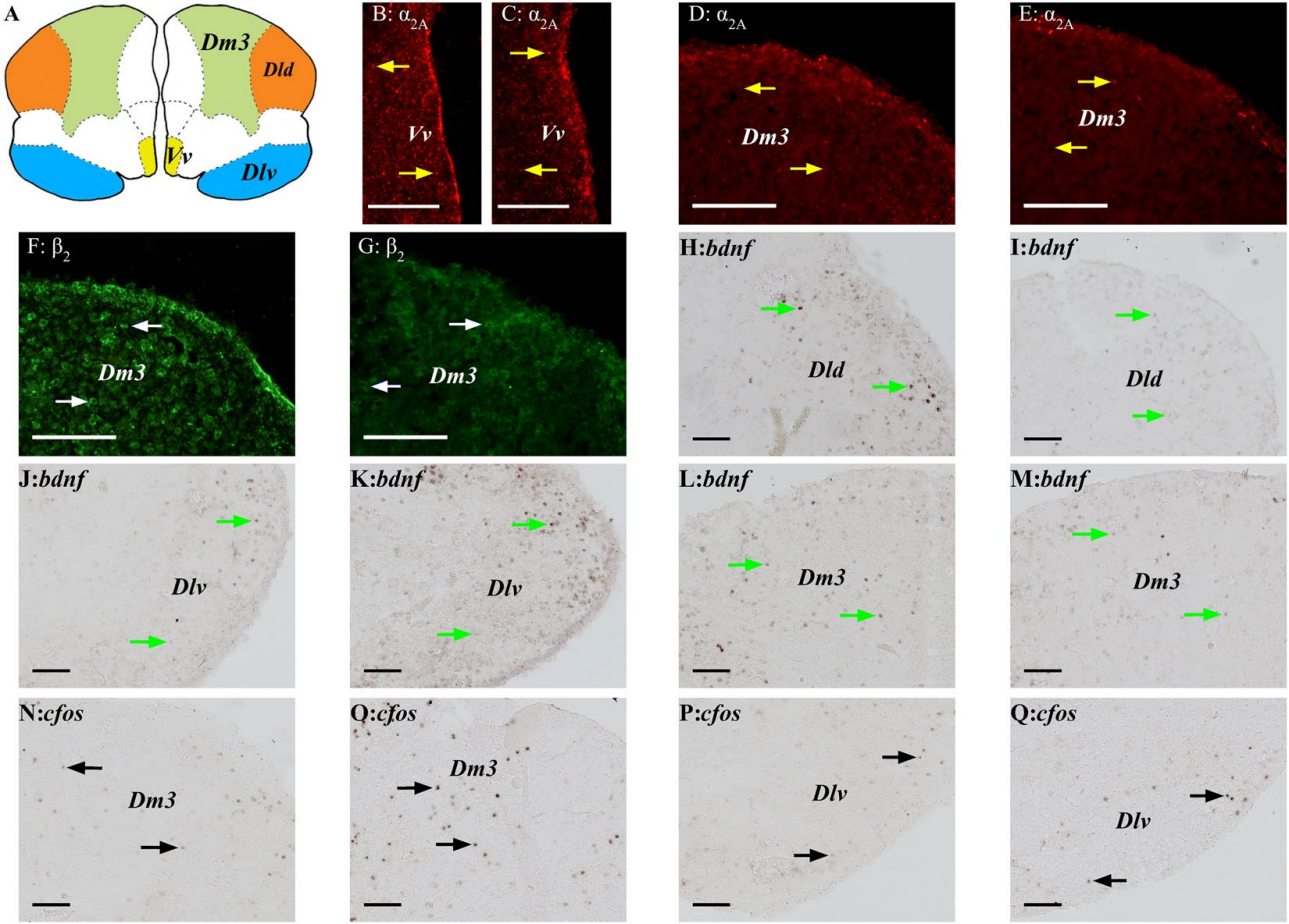
Examples of *in situ* hybridization labelled *bdnf* and *cfos* cells images used in quantification analysis. (**A**) Telencephalic transverse view of Gilthead seabream depicting the location of the dorsal (Dld) and ventral (Dlv) dorsolateral pallium, the dorsomedial pallium subdivision 3 (Dm3) and the ventral part of the ventral telencephalon (Vv). Examples of telencephalic expression of markers at basal levels or in response to acute stress, or in response to treatment (*i*.*e*. control *vs*. early life stress; ELS). ELS 𝛼_2*A*_ expression in the Vv at basal (**B**) and post-acute stress (**C**). Control 𝛼_2*A*_ expression in the Dm3 at basal (**D**) and post-acute stress (**E**). Basal β_2_ expression in the Dm3 in ELS (**F**) and control (**G**). Basal *bdnf* expression in the Dld in control (**H**) and ELS (**I**). Control *bdnf* expression in the Dlv at basal (**J**) and post-acute stress (**K**). Post-acute stress *bdnf* expression in the Dm3 in control (**L**) and ELS (**M**). Post-acute stress *cfos* expression in the Dm3 in control (**N**) and ELS (**O**). Post-acute stress *cfos* expression in the Dlv in control (**P**) and ELS (**Q**). Arrows indicate stained cells for 𝛼_2*A*_ (yellow), β_2_ (white), *bdnf* (green) and *cfos* (black). The scale bars represent 100 μm.

Please refer to Fig. 5 for examples of adrenergic receptors, *bdnf* and *cfos* labelled cells which were used in the quantification analysis.

## Discussion

We found clear region-specific differences between fish that had been previously subjected to an early life stress (ELS) regime, compared to control groups at both basal and post-acute stress levels. The ELS fish were characterized by an overall higher brain catecholaminergic signalling (as indicated by expression of adrenergic receptors and overall higher dopaminergic activity in ELS fish), as well as lower *bdnf* and higher *cfos* expression. Taken together, these results show how ELS treatment has long-term consequences in the way individuals respond to their environment later in life. Interestingly, these differences appear to be mainly related to catecholaminergic systems, since we found no differences in cortisol or serotonergic activity between treatment groups. This is surprising, since both serotonin and cortisol regulate the hypothalamic pituitary interrenal (HPI) axis^28–32^, and as expected, both groups react with increased levels/activity to stress. However, we expected that ELS would directly lead to a difference in how treated individuals regulate these systems at both basal and post-stress conditions, similar to what has been reported in mammals^13^. We believe that a more exhaustive analysis of region-specific serotonergic and cortisol signalling is necessary to shed light on possible effects of ELS, particularly in limbic brain areas. Furthermore, in our experiment we chose a mild chronic ELS in order to avoid mortality in fish groups at these sensitive early stages. Therefore, it is possible that a more severe ELS may lead to more dramatic neurobiological changes, including effects on serotonergic and cortisol regulation. However, further studies are necessary in order to elucidate the possible effects of the magnitude of the ELS on the studied neurobiological parameters.

The brain catecholaminergic systems are fundamental in the control of behavioural flexibility through their role in attention, perception and impulse control^20,33–36^. In this context, high levels of dopamine (DA) and noradrenaline (NA) have been associated with increased arousal during stressful situations^37,38^. These processes are regulated by the interaction of catecholamines with a variety of cellular receptors. Amongst these, the brain adrenergic receptors have been strongly associated with stress coping responses (for a review see^39^). The adrenergic receptors are classified into 2 main classes, α (inhibiting) and β (stimulating), which are in turn, divided into several subtypes^39–42^. Adrenergic receptors are found both pre- and postsynaptic acting as auto- or heteroreceptors, respectively. In mice, it has been found that the majority of the adrenergic autoreceptors are of the α_2Α_ type, which inhibit the transmission of NA^42^. In addition, α_2_ receptors acting as heteroreceptors modify the release of other neurotransmitters such as serotonin, glutamate and acetylcholine^39^. Meanwhile, β_2_ auto- and heteroreceptors have a stimulating effect on their target neurotransmitters^42^. Teleostean brain α and β-adrenergic receptors have been shown to have similar characteristics to those of mammals^43–46^. In this experiment, we found that there was an overall higher brain DA activity in ELS fish, as well as higher expression of α_2Α_ in the ventral part of the ventral telencephalon (Vv) and in the dorsomedial pallium subdivision 3 (Dm3) post-acute stress. Unfortunately, we were not able to determine the NA activity, since it was not possible to quantify the NA’s main catabolite MHPG, due to an increase of interacting peaks (*i*.*e*. noise) in the area were MHPG is expressed in the HPLC chromatogram. However, the adrenergic receptors have been found to regulate both NA and DA signalling^42,47,48^ and their expression can therefore be considered to be indicative of catecholaminergic system activity. Specifically, the α_2Α_ receptor has been shown to regulate both brain NA and DA in a region-specific manner. That is, while α_2Α_ receptors appear to exclusively regulate NA transmission in the cortex and the nucleus accumbens, they regulate DA transmission in the basal ganglia and the ventral tegmental area^42,48^. Therefore, it is tempting to speculate that the higher α_2Α_ expression in the Vv may be part of a regulating mechanism in ELS fish to inhibit catecholaminergic signalling. In other words, the overall higher brain DA activity found in ELS fish may have led to a higher expression of the inhibiting α_2Α_ adrenergic receptor in the Vv, as a regulating mechanism to reduce DA signalling. That is, higher activity of this α_2Α_ autoreceptor in local telencephalic DA populations would lead to increase reuptake of DA by the presynaptic DA neuron which will eventually lead to reduced DA signalling. Notably, similarly to what has been shown in the mammalian lateral septum^49^, we have previously proposed that the Vv may be an important area in DA regulation, since we found a 9- and 7-fold higher concentration of DA and DOPAC in the this area, compared to the Dl and Dm^19^. Furthermore, although we did not include this data in the previously aforementioned study (*i*.*e*. Vindas *et al*.^19^), we also found that there was a 2.5- and 3-fold higher concentration of NA in the Vv, compared to the Dl and Dm. However, this data was not previously published but is now available in the supplementary Figure 1S). Taken together, with the present results, this suggest that the Vv, just like its mammalian functional homologue (*i*.*e*. the lateral septum), is an important brain area for catecholamine regulation^9,49^. We here propose that the inhibiting α_2Α_ receptor, which showed a mean overall higher density in the Vv compared to all other interest areas, may have a DA and NA inhibiting role in the Vv, similarly to what Smith and colleagues^50^ have proposed on the role of this receptor in the inhibition of NA release and anxious-like behaviour in the bed nucleus of the stria terminalis. It is however important to point out that in our experiment we measured whole brain DA activity and not region-specific changes. We believe that a more specific analysis is necessary in order to confirm our current hypothesis on the role of the Vv in catecholaminergic systems regulation. Regarding the β_2_ receptor expression in the Dm3, we found the lowest expression in control fish at basal levels. Furthermore, while control fish showed a significant increase in β_2_ post-acute stress, the same was not true for ELS groups. As stated above, the β_2_ receptor has a stimulating effect on neurotransmitter release and it is therefore expected that this receptor would be upregulated allowing for the increased release of neurotransmitters in response to stress. It is particularly interesting that this effect appears to be exclusive to the Dm3 area, since we also found that the α_2Α_ receptor was downregulated post-stress in this area, possibly resulting in an increase of NA release regulating Dm3 function. In other words, we found a downregulation of an inhibiting receptor of neurotransmitter release, along with an upregulation of a stimulating one. The Dm has been proposed to be the functional homologous to the mammalian amygdala, which is very important in stress reactivity^22,24,51^. In agreement with this literature, the Dm has been found to be highly active and regulated in response to different types of stress in several fish species^19–21,52–54^. However, it is hard to pinpoint which subdivision of the Dm in seabream may be the functional homologous to the amygdala, since there are few functional neuroanatomy studies focused on this topic. However, we propose that since the Dm3 is the most complex of the 4 subdivisions^55^ and it showed the highest amount of stress activity in our experiment, it may be the most likely amygdala-like candidate. Notably, in mammals the amygdala has been associated with the direct regulation of the HPI axis, increasing its activity during stressful situations^56^. In this context, it is interesting to note that ELS fish did not display a post-acute stress downregulation of the α_2Α_ receptor in the Dm3 as control fish did, which could potentially lead to an increase HPI axis activity mediated via the Dm. Taken all together, it is possible that the lack of adrenergic receptor response to stress in this area in ELS fish may be part of the first signs in the Dm3 of cumulative stress overloading physiological systems and compromising their ability to react further to stressors (*i*.*e*. allostatic overload^57^) or mediating stress resilience mechanisms. Noteworthy, anxiety has been found to be a long-term effect of ELS in mammals, which appears to be related to increased activity in the amygdala-septal hypothalamic circuit^12^. It would therefore be interesting to study this amygdala-septal hypothalamic circuit in fish subjected to ELS to see if our current results could be indicative of this brain mechanism. Sampling at several time points throughout the ELS fish´s life would also be necessary to better elucidate the specific long-term stress plasticity events.

We found that the transcript abundance of the neuronal plasticity marker brain derived neurotrophic marker (*bdnf*) was overall lower in ELS fish compared to control in the Dm3 and the Dld. The downregulation of *bdnf* has been previously reported to decrease and increase in response to chronic and acute stress, respectively^58^. Furthermore, chronic downregulation of BDNF has been strongly associated with several neurobiological diseases, such as depression-like states^59^. In this context, it appears that the ELS treatment had a long-term downregulating effect on 2 brain areas associated with memory, learning, navigation and emotional reactivity (*i*.*e*. the Dl and the Dm)^23,24^. Since *bdnf* expression has been strongly associated with promoting neurogenesis, cell survival, and the strengthening of learning and memory^60^, this region-specific downregulation implies that ELS fish may have a diminished capacity regarding the aforementioned processes. Notably, unpredictable chronic stress during adolescence has been found to have a context dependent long-lasting effect on memory systems. Specifically, it appears that while rats subjected to unpredictable chronic stress showed an enhancement in working memory to the location of a reward at basal levels, this was completely hindered after exposure to a novel acute stressor^7^. Interestingly, all fish groups responded with an increase in *bdnf* expression to the acute stressor in the Dlv. As mentioned above, an increase in this factor is expected when animals experience acute stress and the fact that this is strongly expressed in this area for both groups, implies that a higher neural plasticity in this area may help cope with this type of stressor. Notably, in a previous experiment we also found a higher expression of this marker in the Dl (as a whole since we did not differentiate between the dorsal and ventral subregions), after exposing fish to crowding stress^19^. The difference we found between the Dld and the Dlv in our current results suggests that these 2 areas may indeed be involved in the regulation of different processes, in agreement with what has been proposed by Broglio *et al*. based on the topography, connections and histochemistry in these 2 subregions^61^. Further studies are needed including the link between BDNF expression in region-specific areas, memory tasks and ELS in order to understand region-specific neuronal function and the positive and negative effects of ELS in vertebrates.

Neuronal activity can often be characterized by quantifying the expression of immediate early genes^62–64^, such as *cfos*. We found that *cfos* mRNA transcript abundance post-acute stress was higher in ELS fish in both the Dlv and the Dm3. It is important to point out that an increase in *cfos* abundance may reflect either inhibition or excitation of specific neuronal circuitry within each nucleus. In other words, neuronal activity, as inferred by *cfos* transcript abundance, may be indicative of a stimulating or an inhibiting neuron^62^. In this context, the increase *cfos* abundance in ELS fish found in the Dm3 may be directly associated with the higher activity of inhibiting neurons associated with catecholamine regulation, particularly considering the overall elevated expression of both adrenergic receptors in this area. Unfortunately, we were unable to obtain *cfos* levels at basal conditions. Therefore, we cannot confirm that this activation pattern was also present in ELS fish at basal conditions, as it is suggested from our results on the expression of adrenergic receptors. Further studies should be focused on quantifying the expression of early activity genes at both basal and post-acute stress conditions in order to elucidate the overall activity pattern in ELS fish as compared to control individuals.

In conclusion, we found that seabream that experienced an ELS regime display long-term neurobiological brain-region specific effects as juveniles (approximately 5 months after ELS). These results illustrate how neuronal populations within the telencephalon show a distinct regulation to the same stimuli, which may be associated with the control of stress coping responses, in agreement to results obtained in mammalian studies regarding effects of ELS in limbic areas^9,12,13^. In this context, physiological and behavioural responses represent trade-offs from life history strategies and should be viewed/interpreted in a context dependent manner^7,65^, as exemplified by the enhancing and inhibiting effect of unpredictable chronic stress on working memory on rats reported by Chaby *et al*.^7^. We here found that ELS fish showed signs suggesting allostatic overload, which is in contrast to what we found for ELS salmon in our previous studies^32,66^. This may be due to species-specific differences, context-dependent effects to different stressful stimuli or to the exact period in which the ELS regime was given, since it was done at a later age in salmon. We hope that future studies will be focused towards better understanding of allostatic processes and both the possible negative and positive consequences of early life stress in a context- and species-specific dependent manner. Importantly, our data presented here corroborates that the Vv is functionally homologous to the lateral septum, particularly regarding catecholamine regulation.

## Methods

### Ethical permits

All experiments were performed in accordance with the protocol 255332 approved by the HCMR Institutional Animal care and use committee in accordance with Greek (PD 56/2013) and EU (Directive 63/2010) laws and regulations on the use of animals in scientific research. Furthermore, the laboratories of the Hellenic Centre for Marine Research are certified for the breeding and use of animals for scientific purposes (EL-91-BIOexp-04).

### Experimental animals and facilities

Batches of fertilized eggs were obtained from a private fish farm and transferred to the installations of the Institute of Marine Biology, Biotechnology and Aquaculture, HCMR (Heraklion, Crete). Larval rearing was performed applying the pseudogreen water technique^67^ in 500 L conical tanks, where both hatching and rearing took place starting with an initial density of 100 eggs/L.

Tanks were coupled to a biological filter and were initially filled with filtered seawater from a deep well. Water, during embryogenesis, egg hatching and at the autotrophic larval stage, was re-circulated from the bottom of the tank through the biological filter at a rate of 10% per h and was progressively increased to 50% per h. Following first feeding the water renewal in the tanks was set to 20% per h and was gradually increased to 70% at the end of the experimental period. Aeration was provided by means of a wooden diffuser located in the middle of the tank at a rate of 150–200 ml/min.

Larvae were held during the whole experimental period at mean (±SD) water temperature of 18 ± 1.6 °C, dissolved oxygen levels of 7.2 ± 0.8 mg/L, 36‰ salinity and a 7.9 ± 0.3 pH.

During hatching and until mouth opening, tanks were kept in complete darkness while a constant light photoperiod was applied during the rest of the larval rearing.

During rearing, feeding was based on daily supplementation of zooplankton (enriched rotifers and Artemia nauplii) and also phytoplankton for a period of 2 weeks. After 30 days post- hatching (dph) feeding was based on Artemia nauplii and artificial diets (INVE SA).

After 5 dph an air-blower skimmer was used to keep the water surface free from any oily film to ensure normal swim bladder inflation. The larvae rearing period lasted 60 days. Immediately after, the fish were moved into weaning tanks (volume: 10 m^3^ each) and kept until the end of the experiment (*i*.*e*. when juveniles reached approximately 5.5 g) under similar husbandry conditions. In particular, the water from a deep well was of constant temperature (19 ± 1 °C) while maintaining a natural photoperiod. Feeding was based on artificial diets (INVE SA) that was delivered to satiation with automatic distributors and later with demand feeders.

### Experimental procedure

Batches of larvae (of the same genetic origin) were exposed at two different developmental phases (first feeding at 9 days post-hatching; FF and formation of all fins to melanophores at 44 days post-hatching; AF), to an early life stress (ELS) protocol, as developed and described by Tsalafouta *et al*.^68^, for European sea bass, and Sarropoulou *et al*.^69^, for gilthead sea bream. Briefly, the stress protocol consisted of applying randomly 2 out of 4 stressors on a daily basis for a period of two weeks. The stressful stimuli were, lights on/off for 30 min during the night/day, respectively, exposure to blue or red spectrum for 30 min, high aeration for 90 s and exposure to a novel object for 30 min (large sized LEGO of intense red and green colour). In addition, a control group (*i*.*e*. not subjected to the stress regime) was included in the experimental design. All experiments were run in duplicate tanks.

### Sampling protocol

At the end of the experiment (when fish had reached approximately 5.5 g), fish were sampled at basal and acute stress conditions. Individuals were caught by either netting them straight from holding tanks (*n = *20/treatment) or after subjecting them to 10 min crowding, followed by 5 min of chasing with a net and a 1 min air exposure (*n* = 20/treatment). Immediately after netting or after the acute stress tests, individuals were sampled in 2 different ways: (1) fish were deeply anaesthetised with 2-phenoxyethanol (500 ppm; Merck, 807291) and fixed by vascular perfusion with 4% paraformaldehyde (PF) in 0.1 M Sørensen’s phosphate buffer (PB; 28 mM NaH_2_PO_4_, 71 mM Na_2_HPO_4_, pH 7.2). The brains were dissected out and post fixed in fresh 4% PF in Sørensen’s PB for 16 h at 4 °C. The tissue was washed three times 20 min in PB, cryopreserved overnight in 25% sucrose in PB at 4 °C, embedded in Tissue-Tek OCT-Compound (Sakura Fintek) and rapidly frozen in dry ice-cooled isopentane (2-methyl butane; Sigma–Aldrich, Deisenhofen, Germany) at ≈−35 °C and stored at −80 °C until sectioning for immunohistochemistry (*n* = 4/treatment) or *in situ* hybridisation (*n = *6/treatment). (2) Fish were euthanized with an overdose of 2-phenoxyethanol which rendered them completely motionless (no opercular movement) within 10 s of immersion. Fish were then decapitated and the brain was carefully excised within 2 min. Brains were wrapped in individually marked aluminium foil packets, snap-frozen in liquid nitrogen and stored at −80 °C for later analysis of monoamine neurochemistry (*n* = 10/treatment). Trunks were collected and stored at −20 °C for cortisol analysis.

### Cortisol radioimmunoassay

Samples were partially thawed on ice, homogenized in 5× (w/v), ice-cold, phosphate-buffered saline (pH 7.4) and cortisol was extracted by adding 3 mL of diethyl ether to 2 × 250 μl of homogenate. The liquid phase of the extract was frozen by placing the tubes at −80 °C. Thereafter, the combined diethyl ether layer was taken and transferred into a new tube. The tubes were then placed in a water bath at 45 °C for 1 h and then kept at room temperature for an additional 3 h to ensure complete ether evaporation. Samples were then reconstituted in 250 μl of an enzyme immunoassay buffer and cortisol was measured using commercial enzyme immunoassay (EIA) kits (Cayman Chemical, MI, USA)^70^.

### Monoaminergic neurochemistry

Frozen brains were homogenized in 4% (w/v) ice cold perchloric acid (PCA) containing 0.2% EDTA and 3,4-dihydroxybenzyl amine hydrobromide (DHBA, 40 ngml^−1^) as an internal standard using a Potter–Elvehjem homogenizer. After spinning samples for 10 min at 15.493 rcf and 4 °C, the supernatant was analysed by means of high-performance liquid chromatography (HPLC). The HPLC methodology were performed as described in Vindas *et al*.^66^.

### Immunohistochemistry (IHC)

Brains were cut in a cryostat (Leica, CM1500, Wetzlar, Germany) and serial coronal 20 µm sections, spaced 80 µm apart, were mounted on gelatin-coated slides and stored at −80 °C until further analysis. Following, sections were air-dried for 1.5 h and rehydrated with phosphate-buffered saline (PBS; 10 mM). Non-specific protein binding sites were blocked with 0.15% normal horse serum (Vector Laboratories, Burlingame, CA, USA) with 5% bovine serum albumin (BSA; Sigma–Aldrich, Deisenhofen, Germany) in 0.5% Triton X-100 in PBS (PBS-T) for 30 min. Sections for both basal and post-stress samples were then incubated together with primary antibodies for β_2_ adrenergic receptors (dilution 1:50; rabbit polyclonal antibody; Santa Cruz Biotechnology, H-20, sc-569) and α_2A_ adrenergic receptors (dilution 1:70; goat polyclonal antibody, Santa Cruz Biotechnology, C-19, sc-1478) in 0.7% blocking solution in PBS-T at 4 °C overnight. Secondary antibodies, donkey anti-rabbit Alexa Fluor® 488 and donkey anti-goat Alexa Fluor® 568 (dilution 1:400; life technologies, catalogue No A21206 and A11057, respectively), diluted in PBS-T, were also added simultaneously for 2.5 hours at RT in the dark. Finally, sections were thoroughly rinsed, mounted on slides, and left to dry before cover slipping with antifade Mowiol mounting medium (Mowiol® 4–88; Sigma-Aldrich, catalog No 81381).

### *In situ* hybridisation (ISH)

The ISH for *bdnf* and *cfos* transcript abundance was conducted on adjacent sections for control and FF groups only. Note that due to logistical reasons, we were not able to obtain samples for ISH of AF fish. Adjacent transversal 12-µm sections were cut using a Leica CM 1850 cryostat (Leica Microsystems, Wetzlar, Germany), collected on SuperFrost Ultra Plus glasses (Menzel Glaser) and dried at 65 °C for 10 min. Digoxigenin-labeled riboprobes were prepared using a digoxigenin (DIG)-RNA labelling mix in accordance with the manufacturer’s instructions (Roche Diagnostics, Mannheim, Germany). Forward GGCTCGAGTTCATTCTCGCT and reverse GTCGTTGCTGTTGCTTCCTC and forward GGACTCCACAGAGTGGTGGT and reverse CCAGCCAATCTTCTTTTTGC primers were used to clone the *cfos* and *bdnf* probes, respectively. The quality and quantity of the synthesised riboprobes were assessed by agarose gel electrophoresis. Pre-treatment and treatment of sample for ISH was conducted as specified by^71^. Note that sections for both basal and post-stress samples were processed together in order to establish possible differences to both treatment (ELS *vs*. control) and conditions (basal *vs*. post-stress).

### Image analysis: quantification of labelled cells

#### IHC

Adrenergic receptor immunostaining was determined within four telencephalic areas: the dorsal (Dld) and ventral (Dlv; *i*.*e*. both the Dlv1, Dlv2 and Dlv3 subdivisions were pooled together here) parts of the dorsolateral pallium, the third subdivision of the dorsomedial pallium (Dm3) and the ventral part of the ventral telencephalon (Vv). These areas were identified using the gilthead seabream stereotaxic atlas by Muñoz-Cueto *et al*.^55^. Note that the Dm in seabream is divided into several subpopulations (from 1 to 4) due to an increase in complexity (with the Dm3 being the most developed subdivision) compared to other teleost fish species^55^. Preliminary assessments led to the focus areas to be quantified.

For the quantification of adrenergic receptors, the corrected mean density (CMD) for each region of interest was estimated, using the ImageJ software (v. 1.46r, Wayne Rasband, National Institutes of Health (NIH)), according to the following formula:

$${\rm{CMD}}={\rm{Mean}}\,{\rm{Density}}\,{\rm{of}}\,{\rm{selected}}\,{\rm{area}}-{\rm{Mean}}\,{\rm{Density}}\,{\rm{of}}\,{\rm{background}}\,{\rm{readings}}$$

CMD counts of adrenergic receptor labelling were obtained for at least 3 sections per region of interest per animal, while scanning through 20 μm of the tissue section thickness, using a Nikon Eclipse E800 fluorescence microscope at 20× magnification with the aid of a 3CCD Sony DXC-950P camera connected to a PC (Microsoft Windows XP).

#### ISH

Visual quantification of *cfos* and *bdnf* labelled cells was conducted by the use of a light microscope (Optika B-150 POL-B). The number of labelled cells was obtained for each region for both lobes from each slice (in which interest areas were found) in order to calculate an average for each sample corrected by the number of slices (since the number of slices for each interest areas varied between samples). The average number of labelled cells in each area for each fish was used in the statistical analysis.

### Statistical analyses

Two-way analysis of variance (ANOVA) was used to compare cortisol levels, monoaminergic neurochemistry and the IHC and *bdnf* ISH quantified data, with treatment (ELS *vs*. control) and conditions (basal *vs*. acute stress) as independent variables. Models were assessed by their capacity to explain the variability and interaction effects between treatment and conditions were accepted or rejected according to total model “lack of fit” probabilities (provided by the ANOVA model). Significant effects were followed by a Tukey–Kramer honestly significant difference (HSD) post-hoc test. Before final acceptance of the model, diagnostic residual plots were examined to ensure that no systematic patterns occurred in the errors (*e*.*g*. fitted values *vs*. observed values and q-q plots). When necessary values were log transformed (base 10), for cortisol, 5-HT, 5-HIAA, DA, NE, α_2Α_ (in the Dld) and β_2_ (in the Dld). One sample from the FF group was lost during the tissue processing procedures and is therefore missing in the neurochemistry and *cfo*s analysis. In addition, one 5-HT value in the FF post-stress group was determined to be an outlier by use of the Grubbs test and was therefore omitted from further analysis. A Student’s *t* test was conducted to determine significant differences of *cfos* expression between post-stress FF and control groups.

### Data Availability

All relevant data are within the paper and its supplementary material.

## Electronic supplementary material

Supplementary Information

Supplementary Data set
