# Supplementary material for: Early life stress induces long-term changes in limbic areas of a teleost fish: the role of catecholamine systems in stress coping
Source: Sci Rep. 2018 Apr 4;8:5638. doi: 10.1038/s41598-018-23950-x (PMC5884775; doi:10.1038/s41598-018-23950-x)
Supplement: Supplementary file 1 — Supplementary Information [file 41598_2018_23950_MOESM1_ESM.pdf]

Early life stress induces long-term changes in limbic areas of a teleost fish: the role of catecholamine systems in stress coping

Marco A Vindas, Stefanos Fokos, Michail Pavlidis, Erik Höglund, Sylvia Dionysopoulou, Lars O.E. Ebbesson, Nikolaos Papandroulakis, Catherine R. Dermon

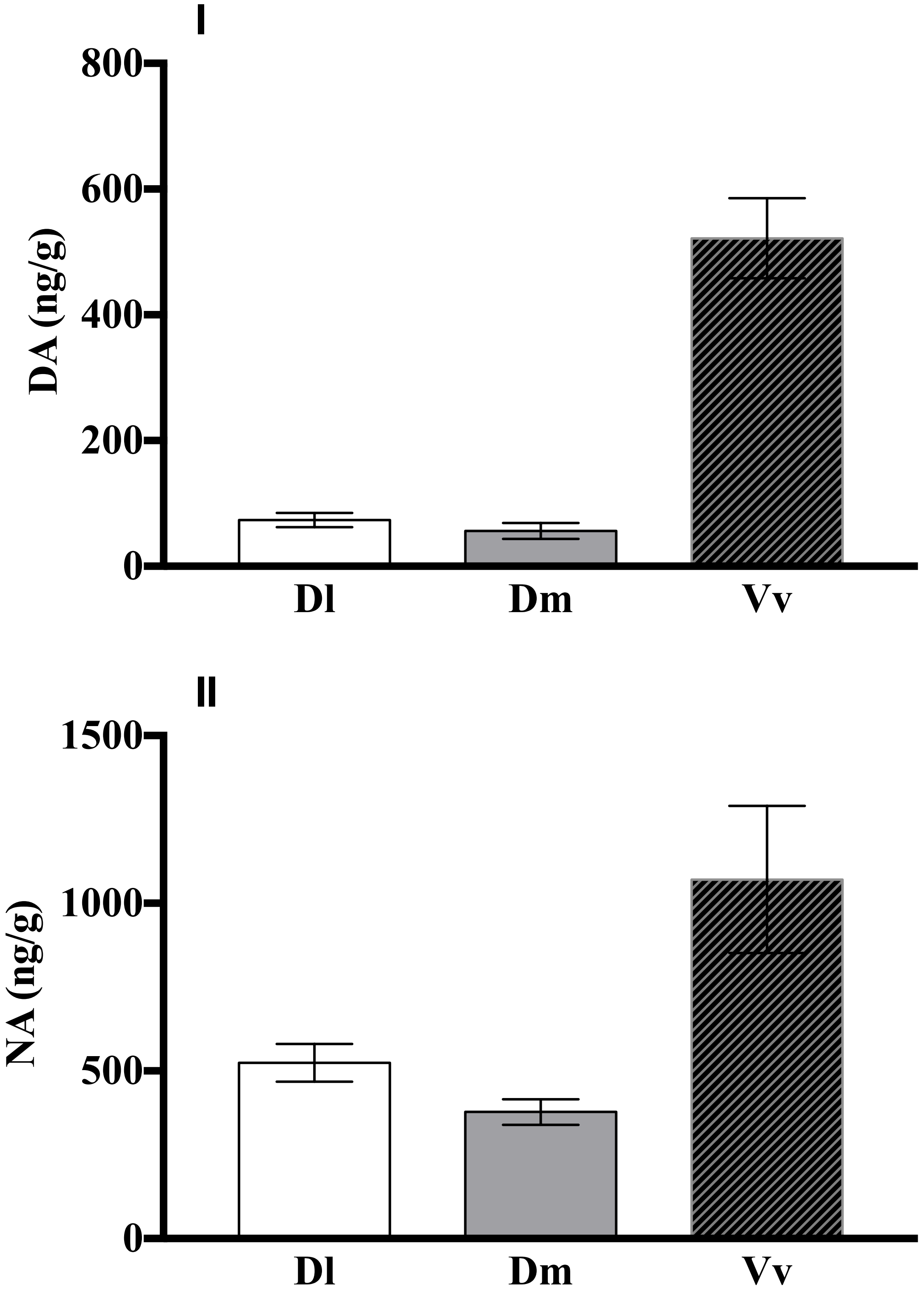

Figure 1S: Dopamine and Noradrenaline in limbic areas. Mean (SEM) concentrations of dopamine (DA; I) and noradrenaline (NA, II) in the dorsolateral pallium (Dl), dorsomedial pallium (Dm) and ventral part of the ventral telencephalon (Vv) of Atlantic salmon. The data for DA neurochemistry has previously been published in Vindas et al. (2017). *J Exp Biol* **220**, 1524-1532. However, even though the data for NA is from the same experiment, this has not been previously published. For details about experimental methodology and results for this experiment please refer to the aforementioned publication.

Supplementary Table 1 Mean (± SEM) concentration of cortisol, the neurotransmitter serotonin (5-HT), its main catabolite 5-HIAA, the neurotransmitter dopamine (DA), its main catabolite DOPAC and the neurotramsitter noradrenaline (NA), as well the 5-HIAA/5-HT and the DOPAC/DA ratios for control or previously exposed to an early life stress regime seabream at either the first feeding (FF) or the all fins (AF) stage at both basal and after acute stress conditions. Two-Way ANOVA statistics for effect of treatment, stress and the interaction between treatment and stress (if it was maintained in the model which was indicated by "lack of fit" analysis), are given for each variable.

	Basal			Stress			ANOVA		
	Control	FF	AF	Control	FF	AF	Treatment	Stress	Interaction
Cortisol	3.74 ± 0.51	3.18 ± 0.27	3.37 ± 0.29	15.01 ± 1.3	15.92 ± 0.05	13.15 ± 1.21	$F_{(3,56)} = 0.61$, $p = 0.55$	$F_{(3,56)} = 392$, $\boldsymbol{p < 0.001}$	------
5-HT	121.5 ± 5.79	133.3 ± 12.7	114.9 ± 6.3	135.8 ± 7.9	125.8 ± 12.5	134.3 ± 8.3	$F_{(3,54)} = 0.5$, $p = 0.61$	$F_{(3,54)} = 3.53$, $p = 0.06$	------
5-HIAA	5.4 ± 0.33	5.22 ± 0.78	4.79 ± 0.54	6.94 ± 0.9	7.57 ± 0.81	6.22 ± 0.6	$F_{(3,55)} = 0.8$, $p = 0.46$	$F_{(3,55)} = 10.6$, $\boldsymbol{p = 0.002}$	------
5-HIAA/5-HT	0.045 ± 0.003	0.039 ± 0.004	0.042 ± 0.004	0.05 ± 0.004	0.055 ± 0.004	0.046 ± 0.003	$F_{(3,54)} = 0.47$, $p = 0.63$	$F_{(3,54)} = 6.3$, $\boldsymbol{p = 0.02}$	------
DA	16.3 ± 0.59	18.1 ± 0.1	15.6 ± 1.32	17.7 ± 1.05	17.6 ± 0.95	17.9 ± 1.09	$F_{(3,55)} = 0.62$, $p = 0.54$	$F_{(3,55)} = 1.7$, $p = 0.19$	------
DOPAC	1.51 ± 0.22	2.75 ± 0.25	2.04 ± 0.24	2.22 ± 0.2	2.36 ± 0.18	2.51 ± 0.2	$F_{(5,53)} = 5.1$, $\boldsymbol{p = 0.009}$	$F_{(5,53)} = 2.2$, $p = 0.14$	$F_{(5,53)} = 3.4$, $\boldsymbol{p = 0.04}$
DOPAC/DA	0.09 ± 0.01	0.15 ± 0.01	0.13 ± 0.01	0.12 ± 0.01	0.14 ± 0.01	0.14 ± 0.01	$F_{(3,55)} = 5.9$, $\boldsymbol{p = 0.005}$	$F_{(3,55)} = 0.64$, $p = 0.43$	------
NE	24.1 ± 2.78	24 ± 2.86	19.4 ± 2.12	21.3 ± 1.84	20 ± 0.85	25.1 ± 3.1	$F_{(3,55)} = 0.1$, $p = 0.9$	$F_{(3,55)} = 0.02$, $p = 0.91$	------
